# Complete Genome Sequence of Swine Hepatitis E Virus Prevalent in Southwest China

**DOI:** 10.1128/genomeA.00090-14

**Published:** 2014-03-13

**Authors:** Wenhai Yu, Tianwu Ma, Xianchen Zhao, Zhanlong He, Chenchen Yang, Yanhong Bi, Fen Huang

**Affiliations:** a Medical Faculty, Kunming University of Science and Technology, Kunming, China; b Institute of Medical Biology, Chinese Academy of Medical Sciences & Peking Union Medical College, Kunming, China

## Abstract

Hepatitis E virus (HEV) is an important public health concern in the world, especially in developing countries of Africa and Asia, including China. Hepatitis E is recognized as a zoonotic disease, which is transmitted across species, including between humans and swine. HEV is highly endemic in China, but the complete sequence of HEV in southwestern China is lacking. Swine HEV strain KM01 was isolated from a village in rural Kunming, Yunnan province, China, where swine are housed with humans. Here, we report the complete genome sequence of the swine HEV strain KM01. The sequence and phylogenetic analyses reveal that swine HEV is closely related to the strain isolated from Xinjiang (CHN-XJ-SW13). The genome of the KM01 strain will facilitate further study of HEV molecular epidemiology and genetic diversity in China.

## GENOME ANNOUNCEMENT

Hepatitis E (HE) is an acute self-limiting disease in humans, causing particularly high mortality in pregnant women (1). HE virus (HEV) is a zoonotic virus, which is transmitted across species between humans, swine, boars, deer, chicken, and rabbits (2–5). HEV antibodies have been found in pigs, rats, cats, and cattle (6–9), with pigs being identified as important reservoirs. Evidence shows that veterinarians working with swine are at increased risk for HEV infection (7). HEV has been found in commercially available pig livers (3, 10). HEV is highly endemic in China (11), but epidemiology data are rare in southwestern China, in the Yunnan province, where raw pork is consumed frequently during Spring Festival.

We report here the complete genomic sequence of the first strain of swine HEV from Yunnan province, China, strain KM01. Swine fecal samples collected from a village near Kunming city were tested for HEV RNA by reverse transcription-nested PCR (RT-nPCR) using degenerate primers. Overlapping fragments covering the complete genome were subsequently amplified separately from seven fragments. The extreme 5′ and 3′ ends of the viral genome were amplified using the Rapid Amplification of cDNA Ends (12) technique. The complete genomic sequence was assembled and analyzed using the MegAlign software.

Excluding the poly(A) sequence, the complete genome of the KM01 strain of swine HEV is 7,240 bp in length, and the G+C content is 55%. The genomic organization of the swine HEV is similar to those of other genotype 4 HEVs, with a 5′-untranslated region (13) (nucleotides [nt] 1 to 26), followed by open reading frame 1 (ORF1) (nt 27 to 5150), ORF2 (nt 5147 to 7171), ORF3 (nt 5175 to 5519), and the 3′-untranslated region (UTR) (nt 7172 to 7264). There is 3 nt of overlap between ORF1 and ORF2. The ORF3 of the KM01 isolate completely overlapped within ORF2, which was similar to most genotype 4 isolates.

KM01 shares approximately 74.9%, 74.1%, 75.4%, and 96.4% nucleotide sequence identities across the entire genome with the genotype 1, 2, 3, and 4 HEVs, respectively. Phylogenetic analysis reveals that swine HEV is similar to genotype 4 HEV infecting swine in Xinjiang and humans in Wuhan, China, which indicates that this swine strain of HEV is zoonotic and capable of infecting across species barriers to humans.

### Nucleotide sequence accession number.

The complete genome sequence of the swine HEV strain was deposited in the GenBank database under the accession no. KJ155502.
